# Fabrication of Ternary MoS_2_/CdS/Bi_2_S_3_-Based Nano Composites for Photocatalytic Dye Degradation

**DOI:** 10.3390/molecules28073167

**Published:** 2023-04-02

**Authors:** Asif Nazir, Muhammad Suleman Tahir, Ghulam Mustafa Kamal, Xu Zhang, Muhammad Bilal Tahir, Bin Jiang, Muhammad Safdar

**Affiliations:** 1 Institute of Chemistry, Faculty of Natural and Applied Sciences, Khwaja Fareed University of Engineering and Information Technology, Rahim Yar Khan 64200, Pakistan; 2 Institute of Chemical and Environmental Engineering, Khwaja Fareed University of Engineering and Information Technology, Rahim Yar Khan 64200, Pakistan; 3 Department of Chemical Engineering, University of Gujrat, Gujrat 50700, Pakistan; 4 Optics Valley Laboratory, Wuhan 430074, China; 5 State Key Laboratory of Magnetic Resonance and Atomic Molecular Physics, Key Laboratory of Magnetic Resonance in Biological Systems, National Center for Magnetic Resonance in Wuhan, Wuhan Institute of Physics and Mathematics, Innovation Academy for Precision Measurement Science and Technology, Chinese Academy of Sciences, Wuhan 430071, China; 6 Wuhan National Laboratory for Optoelectronics, Huazhong University of Science and Technology, Wuhan 430071, China; 7 Institute of Physics, Khwaja Fareed University of Engineering and Information Technology, Rahim Yar Khan 64200, Pakistan

**Keywords:** CdS/MoS_2_/Bi_2_S_3_, photocatalysis, photodegradation, organic dye

## Abstract

The synthesis and design of low-cost visible-light-active catalysts for the photodegradation of organic dyes have been regarded as an efficient way to use solar energy in addressing environmental issues. We report the fabrication of MoS_2_/CdS nanoparticles functionalized with Bi_2_S_3_ nanoflakes. The ternary composites of “MoS_2_/CdS/Bi_2_S_3_” were synthesized in situ by a hydrothermal method at different temperatures. The changes in structural, optical, and morphological properties of the synthesized CdS/MoS_2_/Bi_2_S_3_ were explored. The effects of Bi_2_S_3_ on CdS/MoS_2_ were thoroughly studied by performing an X-ray diffractometer (XRD), a scanning electron microscope (SEM), an ultra-violet–visible spectrometer (Uv–vis), and Fourier transform infrared spectroscopic (FT-IR) studies of the nanoparticles. XRD confirms the cubical crystal structure of the nanoparticles. SEM studies possess the modulation in the surface morphology with the tenability in volume ratios of “MoS_2_/CdS/Bi_2_S_3_” composites. It was observed that the bandgaps calculated using absorption measurements could be manipulated from 2.40 eV to 0.97 eV with varying Bi_2_S_3_ in the MoS_2_/CdS nanostructures. FT-IR confirmed the synthesis of “MoS_2_/CdS/Bi_2_S_3_” nanoparticles. On allowing the visible light to fall for 120 min, it was observed that “MoS_2_/CdS/Bi_2_S_3_” degrades the methylene blue up to 90%. The calculated results of “MoS_2_/CdS/Bi_2_S_3_” suggest that the synthesized material could be a strong candidate for photodegradation applications. This research work explains the synthesis of MoS_2_/CdS/Bi_2_S_3_-based nanocomposites for the degradation of dye using a photocatalytic process. The final results show that this catalyst effectively degrades the dye.

## 1. Introduction

Water is necessary for many aspects of life, including growth and development. Safer and healthier drinking water is essential for maintaining public health [1]. Water is frequently used as one of the vital sources of life [2]. Water is a precious resource for many purposes, including domestic, industrial, and agricultural ones. It is also crucial for metabolic processes and cell function [3].

However, as the environment continues to deteriorate, there are various problems related to water pollution that also contaminate other parts of the ecosystem [4]. Furthermore, once polluted, the treatment of water is challenging and expensive, making it almost always impossible [5]. Water and water resources are essential for ensuring that all living species have access to enough food and a fertile habitat [6]. As the human population and economic activity have increased, so has the global need for freshwater [7]. Critical water resources are being placed under increasing pressure due to the adverse impacts of a growing global population, a changing climate, and different consumption habits, leading to widespread water stress in many countries [8]. Due to this, people are becoming more conscious of the urgent need to reduce their water use [9]. While developing nations face severe water pollution problems, industrialized nations still face pollution problems today [10]. Currently, 1.1 billion people are in danger because of a shortage of clean water, and 35% of fatal illnesses associated with water use occur in developing countries [11]. 

The development of technology has led to the introduction of several physical, chemical, and biological pollutants into the water supply. Wastewater includes liquid waste from houses, institutions, and industrial organizations. The pollution of waterways by industrial effluent is a global problem. There are high levels of several organic contaminants, heavy metals, and non-degradable compounds. Effectively eliminating these contaminants from industrial wastewater is of critical importance at present. Successful cleansing processes are required to eliminate these contaminants before they may be thrown away. Treatment methods for industrial wastewater may be divided into three broad categories: chemical, physical, and biological. The most often used technologies may be categorized as pre-treatments, primary treatments, secondary treatments, and tertiary treatments. Primary treatments, regularly used for essential cleaning, are size-based separations utilizing physical procedures similar to sedimentation and filtration. Secondary treatment can include physical, chemical, or biological methods to remove 80–90% of BOD, COD, and TSS from the wastewater. Tertiary treatment is used to further clean the effluent after secondary treatment by removing any harmful or dangerously recalcitrant pollutants (in some instances, this may be as high as 99%).

One of the methods available for efficiently treating wastewater is the use of nanomaterials. Water purification utilizing inexpensive nanoadsorbents and nanofiltration is an innovative use for nanomaterials. In this overview, we look at how nanoparticles are used to clean up industrial wastewater, emphasizing MB removal.

Industrial wastewater must be cleaned of organic dyes using practical, affordable methods that should be sustainable for the environment. The use of nanocomposite photocatalysts has been a very efficient way to treat industrial waste waters.

The use of photocatalysts is the most effective strategy for reducing organic dyes in manufacturing waste. The photocatalytic technique may be used to lessen environmental damage caused by industrial waste. CdS is one of the most promising photocatalysts for its nontoxicity, low cost, and photostability [9,10]. However, because of its large bandgap (2.42 eV), this frequently used CdS has poor photocatalytic effectiveness and is only photocatalytically active when exposed to UV light. Due to the smaller bandgap, visible-light photocatalysis was made possible [11]. However, several investigations have shown that CdS has weak photocatalytic activity. Metal-nonmetal doping is often used to create a defect level in the CdS band that is outlawed to optimize sunlight use [12,13].

The recombination impact of carriers further reduces the photocatalytic efficiency, yet a modest number of flaws are still virtually inadequate to improve the absorption of visible light radiation [14,15]. The reduced CdS (Cds-x) shows excellent visible light absorption and possesses a Cd^2+^ or sulfur vacancy defect. Under xenon-light illumination, it was found that the rate of methylene blue (MB) deterioration was 2.7 times greater than that of pure CdS. CdS-x offers active sites in the prohibited bandgap and maintains the natural structure, in contrast to impurity incorporation.

Although the decreased CdS (CdS-x) had more flaws, substantial photoelectron and hole recombination continued to occur in single-phase CdS, resulting in poor photocatalytic efficiency and hampering its use [16]. Heterostructures have been used to reduce carrier recombination by using semiconductor compounds, the deposition of noble metals, and the modification of carbon materials [17,18].

Nanoparticles are the most common sort of nanostructured material. According to the nanoscale model, nanoparticles are self-existent material units that are entirely isolated from one another by neighboring particles of a uniform size. They may also have a wide range of morphologies [17]. The creation of customized nanoparticles and their optimization for applications in various technological fields, including electronics, biomedicine, and catalysis, have received much attention during the last 20 years.

Nanoparticles fabricated from inorganic phases (metals, alloys, oxides, and composites) are typically preferred because of their enhanced electrical, optical, magnetic, and mechanical capabilities and the increased opportunities afforded by the smaller-than-nanometer-scale coupling between them [18]. Most industrial, environmental, and biological activities are based on aqueous chemistry, which is especially suitable for inorganic nanoparticles.

Furthermore, it has been suggested that nanoparticles could be used to filter out organic dyes from drinking water. Numerous early studies back up the idea that some particles may irreversibly remove one or more organic dyes and metals from industrial wastewater.

The thermal instability of these modified materials is causing a significant decline in photocatalytic activity [19,20]. Due to its thermal stability and capacity to offer catalytic activity, MoS_2_/Bi_2_S_3_ is a favored material. The photocatalytic characteristics of all the produced CdS/MoS_2_/Bi_2_S_3_ heterostructure photocatalysts are superior to those of pure CdS due to improved charge carrier separation efficiency [21,22].

Though, the synthesis technique of CdS/MoS_2_ composites is laborious, and the recycling process is challenging;, still the material becomes more efficient by intimate contact of MoS_2_ on the surface of CdS The photocatalytically active, micron-sized CdS/MoS_2_ composite photocatalysts are currently made using a three-step external source [23,24]. We proposed the loading of Bi_2_S_3_ on the surface of CdS/MoS to synthesize a new composite with further enhanced photocatalytic activity. Through a unique hydrothermal process under embedded sintering conditions has been used in this study to manufacture an in-situ grey CdS/MoS_2_/Bi_2_S_3_ hetero-structure photocatalyst in a single step. Earlier research has revealed its ability to treat high, medium, and low turbidity water. It can also be used as a softening or dewatering agent, so its importance in wastewater treatment cannot be overstated.

When “CdS/MoS_2_/Bi_2_S_3_” is compared with conventional chemical coagulants, it has the following advantages: cost effectiveness, availability, biodegradable sludge, eco-friendliness, low sludge volume, it does not produce harmful by-products, it is easily handled as it is not corrosive, and it does not affect the pH of water. In the light of the above advantages, “CdS/MoS_2_/Bi_2_S_3_” is environmentally friendly and available at a low cost, which can be a good alternative to chemical photocatalysis with a potential application in wastewater treatment in industry. Therefore, using “CdS/MoS_2_/Bi_2_S_3_” as a photocatalyst in the industrial waste strategy to improve water quality will provide the best results.

## 2. Results and Discussion

It was possible to identify the crystal structure using an X-ray diffractometer. A scanning electron microscope (SEM) was used to analyze surface morphology. FT-IR confirms the synthesis of MoS_2_/CdS/Bi_2_S_3_ nanoparticles.

### 2.1. Morphological Studies

**Figure 1:**
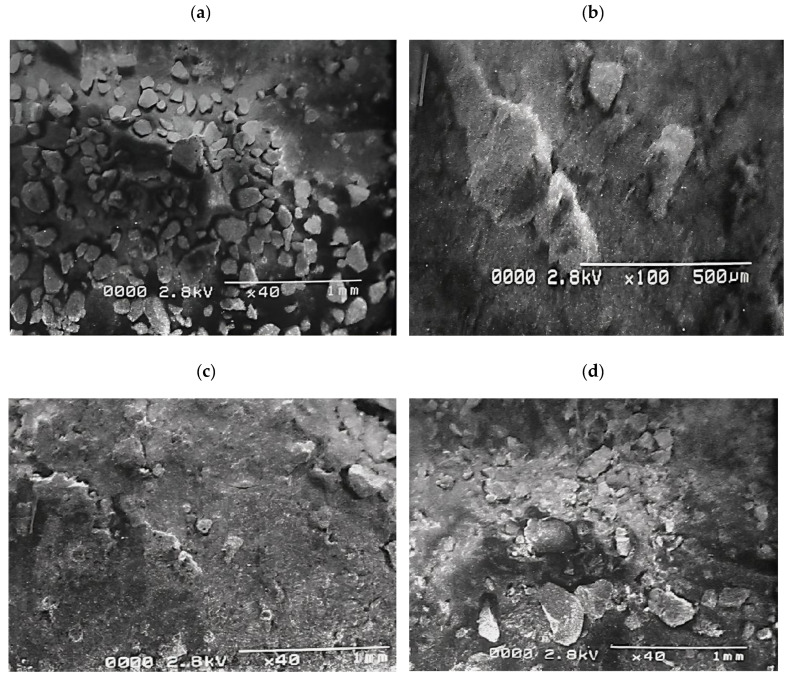

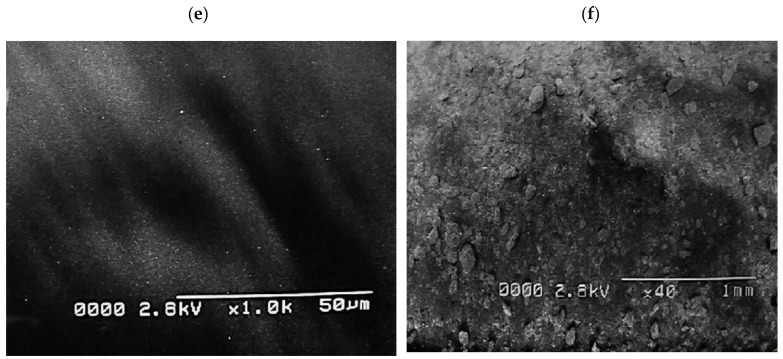
(**a**) CdS, (**b**) MoS_2_, (**c**) CdS/MoS_2_/Bi_2_S_3_ composites (1%/1%/1%), (**d**) CdS/MoS_2_/Bi_2_S_3_ composites (1%/5%/5%), (**e**) CdS/MoS_2_/Bi_2_S_3_ Composites (2%/5%/5%), and (**f**) CdS/MoS_2_/Bi_2_S_3_ composites (0.1%/1%/1%).

The nanoscale MoS_2_ particles are visible and successfully prepared composites of MoS_2_ nanostructure. The morphology of MoS_2_/CdS was characterized using surface morphology as it was created. Figure 1a,b, respectively, show SEM images of CdS on MOS_2_. The CdS, which resembled an olive, was shaped like a nanoflake’s surface [25,26].

MoS_2_ was connected irregularly to the edge of the CdS in the MoS_2_/CdS/Bi_2_S_3_ composite, increasing the catalytic edge sites [27,28].

The brilliance of the orange colour in the MoS_2_/CdS composite decreased when the ratio of MoS_2_/Bi_2_S_3_ increased. The surface-to-volume ratio of nanostructured materials makes assessing their surface highlights a must for several essential applications. Figure 1c shows the CdS/MoS_2_/Bi_2_S_3_ composites (1%/1%/1%), the Figure 1d CdS/MoS_2_/Bi_2_S_3_ composites (1%/0.5%/0.5%), the Figure 1e CdS/MoS_2_/Bi_2_S_3_ composites (2%/5%/5%), and the Figure 1f CdS/MoS2/Bi_2_S_3_ composites (0.1%/1%/1%). The particles of nanosized CdS, MoS_2_, and Bi_2_S_3_ are obviously clear, proposing a successful composite of CdS, MoS_2_, and Bi_2_S_3_ nanostructures. SEM images of complete CdS/MoS_2_/Bi_2_S_3_ nanoparticles demonstrate CdS/MoS_2_/Bi_2_S_3_ nanoparticles.

### 2.2. Structural Studies

**Figure 2:**
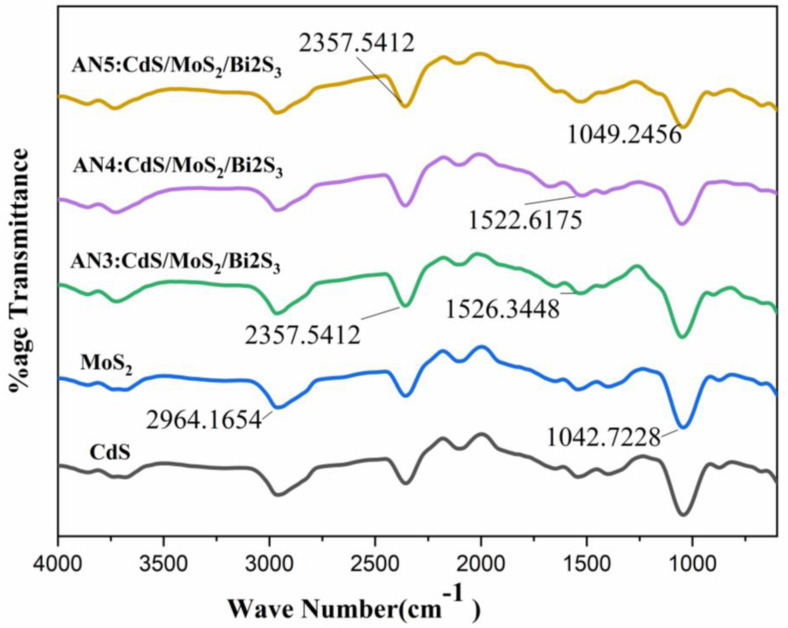
FTIR spectra of CdS, MoS_2_, (AN3)CdS/MoS_2_/Bi_2_S_3_ composite (1%/1%/1%), (AN_4_)CdS/MoS_2_/Bi_2_S_3_ composite (1%/5%/5%), and (AN5)CdS/MoS2/Bi_2_S_3_ composite (2%/5%/5%).

The overlapped FTIR spectra of the synthesized materials have been shown in Figure 2. The FTIR spectra cover an array of 4000 cm^−1^ to 400 cm^−1^. The major peaks are at 3399 cm^−1^, 1624 cm^−1^, 1112 cm^−1^, and 418 cm^−1^. The extending style of the OH group, which is specified by water contents, is designated by an eclectic band at 3399 cm^−1^. The H-O-H bending vibrational mode is responsible for the band about 1624 cm^−1^, which is absorbed by water in the air. The FT-IR spectra of the precursor and product MoS_2_/CdS/Bi_2_S_3_ nanocomposites are also shown in Figure 2. The I.R. bands at 428 cm^−1^ and 508 cm^−1^ are symptomatic of the stretching vibrations of the Mo-S and (S-S)2 bonds [25,29].

Both the stretching vibration of the CdS link at 630 cm^−1^ and the bending vibration of water at 1560 cm^−1^ were both identified. Specifically, the prominent peak between 3200 and 3500 cm^−1^ was associated with the O-H bond vibration [26]. This suggested that photoreduction, rather than adsorption, was the primary interaction mechanism on the MoS_2_/CdS composite, as no new bands appeared following the reaction [30,31].

The absorptions at 1627, 1427, 1112, 875, and 607 cm^−1^ are all ascribed to MoS_2_, although the bands at 3000–2800 cm^−1^ are produced by the stretching of the C-H alkyl stretching band in polyethylene glycol. Finally, FTIR concerns have long established the favorable implementation of MoS_2_ nanocomposites [32].

We examined the FTIR range of the contrived MoS_2_ nanoparticles and shown in Figure 2. Wide-ranging absorption bands were found at 639 cm^−1^, 893 cm^−1^, 1402 cm^−1^, and 1622 cm^−1^ which are accredited to MoS_2_. The band at approximately 483 cm^-1^ is due to the S-S bond. The 931 cm^−1^ band is owed to the S-S bond. The peaks of about 3182 cm^−1^ are distinguishing features of the O-H group [33,34].

Strong hydrogen bonding between the O-H bands at 3170 cm^−1^ causes them to commonly overlap the C–H absorbance. CdS particles showed two C-O stretching bands at 997 cm^−1^ and 653 cm^−1^, and CH_3_ (acetone) bending, whether acute or wide, was papered at 1484 cm^–1^. Impurities such as SO_4_ can be detected as a small absorption peak at 853 cm^−1^. The absorption at 2002–2926 cm^−1^ is recognized as a stretching vibration of carbon and hydrogen.

Along with the peaks at 400–1000 cm^−1^, which are linked with Bi-S stretching vibration, an alternative peak seems to be at 1110 cm^−1^, which is accompanied by C–O, C=O, and C–S bending vibration.

### 2.3. XRD Analysis

**Figure 3:**
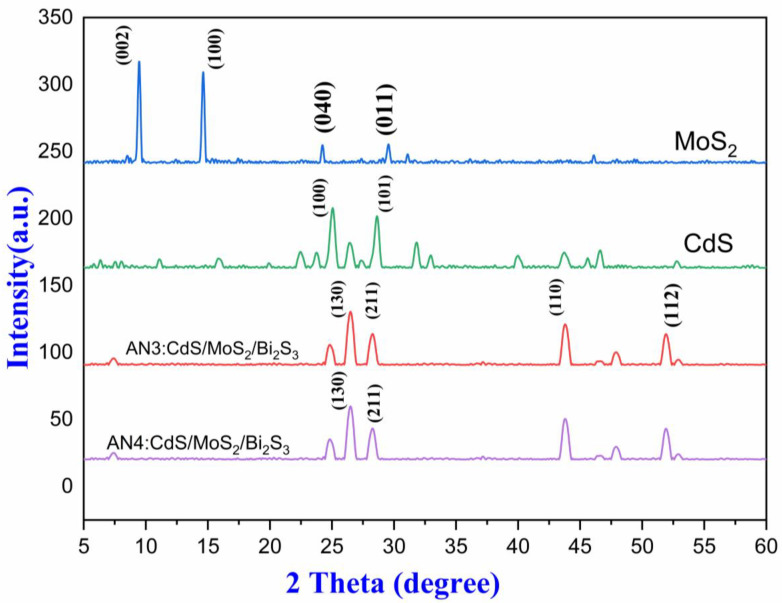
XRD spectra (CdS, AN3, AN4, and MoS_2_).

Since atoms in different crystals release X-rays in different directions. Using an X-ray diffractometer (XRD JDX-3532 JEOL, Tokyo Japan), the crystallinity of as-prepared MoS_2_/CdS/Bi_2_S_3_ was determined. A three-dimensional arrangement of electron density may be indomitable by evaluating the angles and scattering intensities of these beams. This technique may be used to determine crystal structure, chemical bonding, and other similar phenomena. Porous molybdenum disulfide’s XRD pattern can be seen in Figure 3. Porous MoS_2_ was effectively synthesized, as evidenced by peaks at 14.3° and weak peaks at 24.3° and 29.2°. (JCPDS card No. 37-1492).

Furthermore, the XRD spectrum of CdS nanoparticles is presented in Figure 3; peaks at 25.8, 43.2, and 51.3 °C agree with JCPDS 42-1411, representing the triumph for the production. Figure 3 also pertains to AN3 (CdS/MoS_2_/Bi_2_S_3_) 1%1%/1% nanocomposites at 26.9°, 44.7°, and 52.5° [35]. Figure 3 also has to do with AN4 (CdS/MoS2/Bi2S3, 1%:5%:5%) at 25.1°, 27.3°, and 46.1° to sanction the manifestation of the components Cd, S, and Mo in the manufactured nanoparticles and nanocomposites. The crystal configuration of MoS_2_/CdS was deliberated using XRD analysis [36]. The intense diffraction peak of the (101) plane shows that CdS crystals grew in that orientation. The diffraction peak intensity of MoS_2_/CdS was big and crisp, indicating excellent crystallization. The lack of a noticeable shift in the diffraction peak of MoS_2_/CdS composites when compared to CdS revealed that the loading of MoS_2_ rather than doping was the cause. The lack of a diffraction peak in MoS_2_ could be due to its low content [37]. After the photocatalytic reaction, the diffraction peaks did not significantly shift, indicating that the MoS_2_/Cd/Bi_2_S_3_ composite was stable. It is acknowledged that high crystallinity is related to a reduction in crystal defects, which prevents the recombination of photogenerated electrons and holes and improves photocatalytic activity.

### 2.4. UV–Vis Absorbance Spectra

**Figure 4:**
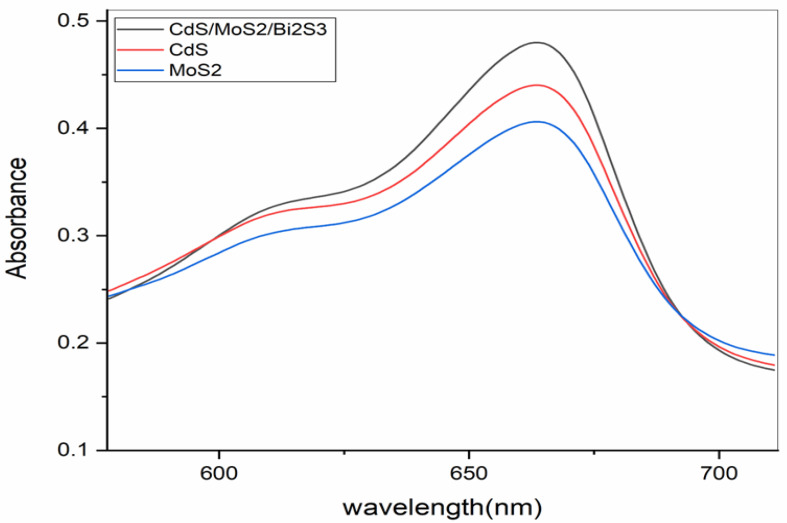
UV-Vis spectrum (CdS/MoS2/Bi_2_S_3_, CdS, and MoS_2_) before degradation.

The optical traits of MoS_2_/CdS nanocomposites were studied using UV-Vis and photocatalytic studies. UV-Vis spectra (for MoS_2_, CdS, and the MoS_2_/CdS nanocomposite) are presented in Figure 4. The UV-Vis absorption of MoS_2_ is very weak, but when CdS is incorporated into MoS_2_ to produce a MoS_2_/CdS nanocomposite, the absorption is enhanced and pushed down to shorter wavelengths. Studies in the UV-Vis range have been used to learn about the optical properties of MoS_2_/CdS nanocomposites. It is important to note that MoS_2_ has weak UV-Vis absorption; however, that UV-Vis absorption is enhanced by a MoS_2_/CdS nanocomposite.

**Figure 5:**
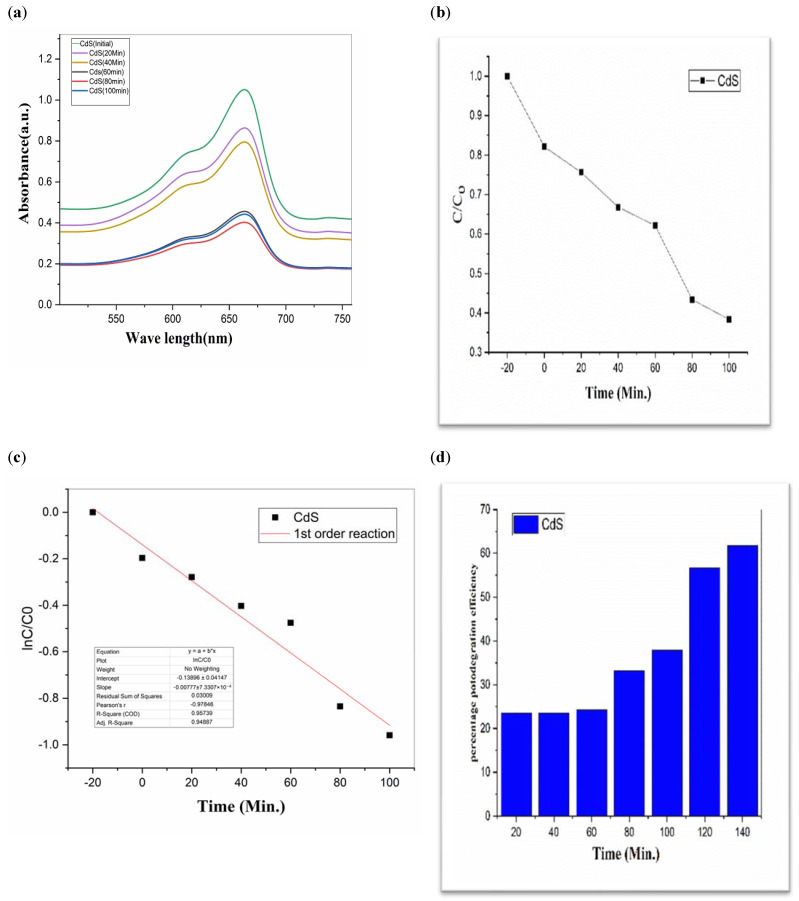
CdS (**a**) graphical representation of degradation of methylene blue; (**b**) C/C_0_; (**c**) lnC/C_0_; (**d**) %age efficiency.

**Figure 6:**
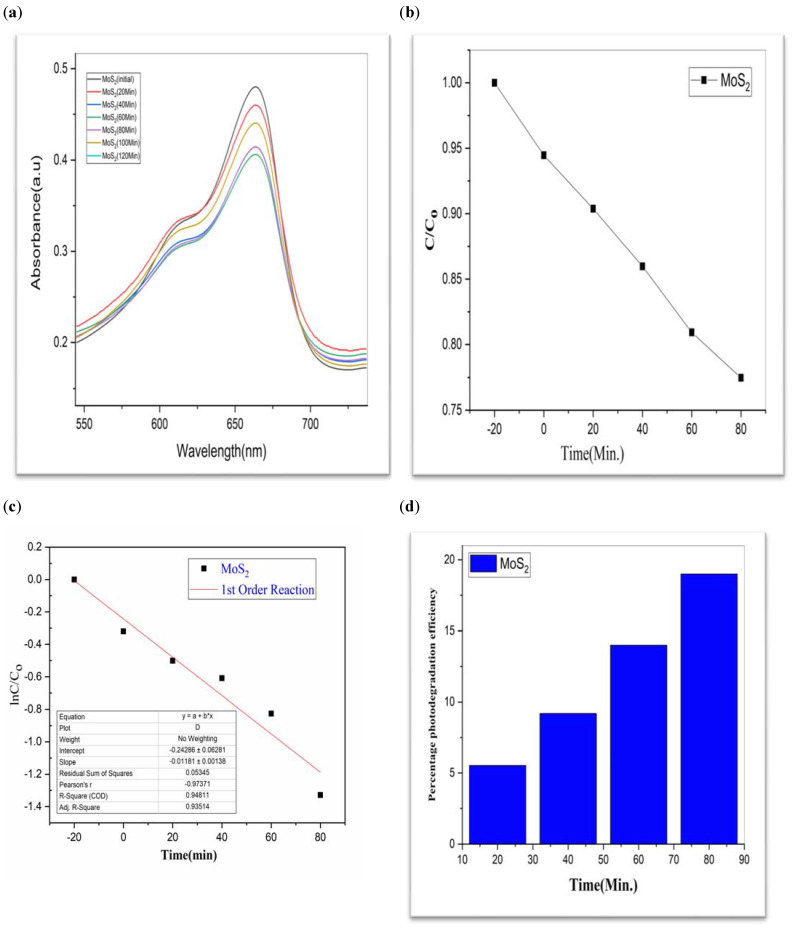
MoS_2_ (**a**) graphical representation of degradation of methylene blue; (**b**) C/C_0_; (**c**) lnC/C_0_; (**d**) %age efficiency.

CdS nanoparticles, as shown in Figure 5a, feature a 3.4 eV bandgap and a maximum wavelength of 454 nm. The absorption spectra in Figure 4 show MoS_2_’s 1.6 eV bandgap [38]. Nanocomposites MoS_2_/CdS have a 2.8 eV bandgap, and their absorption spectra are shown in Figure 6 [36]. Due to the incorporation of MoS_2_, the bandgap energy was raised. The MoS_2_/CdS nanocomposite also exhibited an enhanced absorption curve in the visible spectrum.

The bandgap was found by using the following formula:

$$Eg=\frac{1240}{\lambda }$$

### 2.5. Photocatalytic Activity of CdS/MoS_2_/Bi_2_S_3_

When exposed to ultraviolet (UV) or visible light, the catalyst becomes active, producing free electrons and holes on its surface in addition to hydroxyl radicals. These free radicals oxidize and break down or eliminate organic pollutants. Reacting with the surface, oxygen is found naturally in the air, and water frees electrons trapped in the CdS. Due to its interaction with unstable oxygen free radicals, the O^2-^ anion is formed, further oxidizing the organic molecules (superoxide).

CdS/MoS_2_ is a useful photocatalyst because its surface undergoes many processes necessary for the degradation of organic dyes. The following formula was used to determine the effective photocatalytic degradation of organic dyes.

photocatalytic degradation efficiency = (C_0_ − C/C_0_) × 100

where C_0_ is the dye solution concertation before photoirradiation, and C is the concertation of solutions in suspension after photoirradiation for a given time t [39,40].

**Figure 7:**
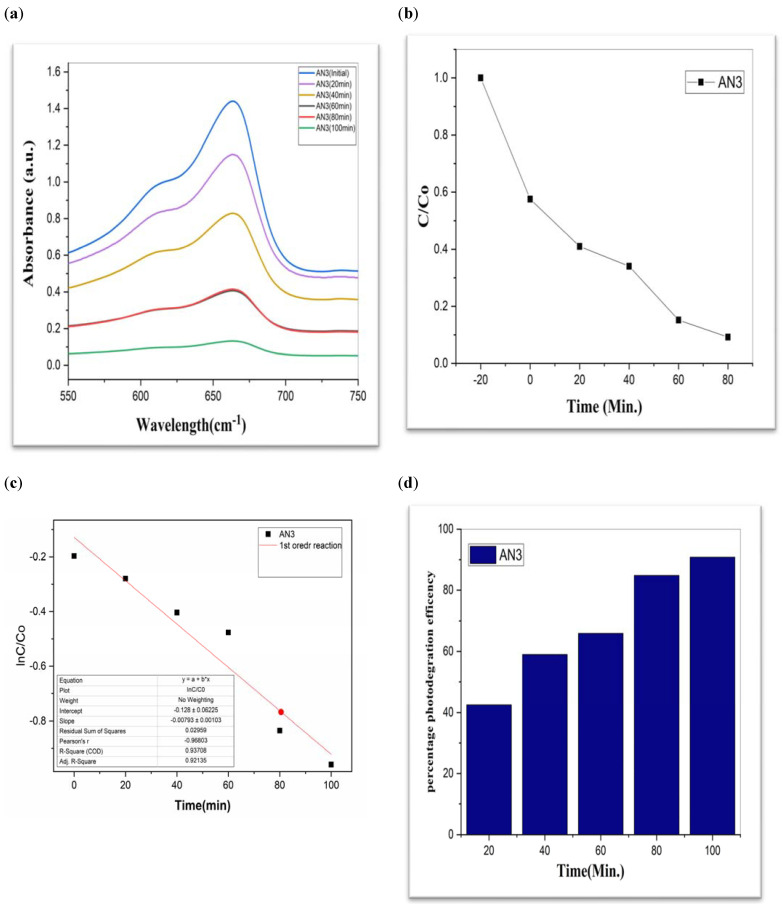
AN3 (**a**) graphical representation of degradation of methylene blue; (**b**) C/C_0_; (**c**) lnC/C_0_; (**d**) %age efficiency.

**Figure 8:**
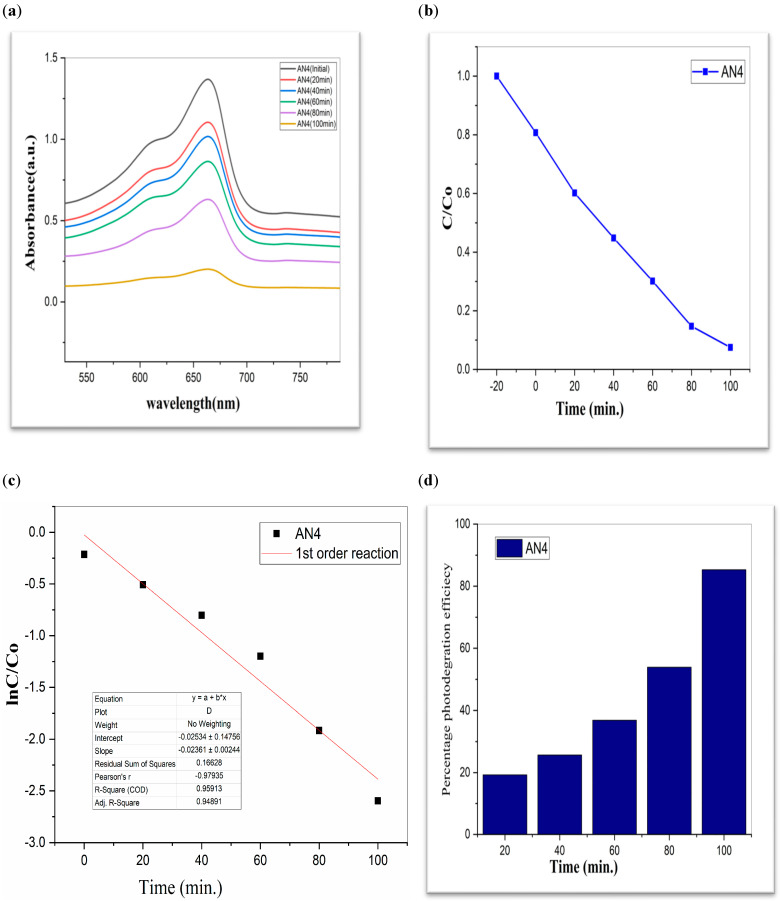
AN4 (**a**) graphical representation of degradation of methylene blue; (**b**) C/C_0_; (**c**) lnC/C_0_; (**d**) %age efficiency.

**Figure 9:**
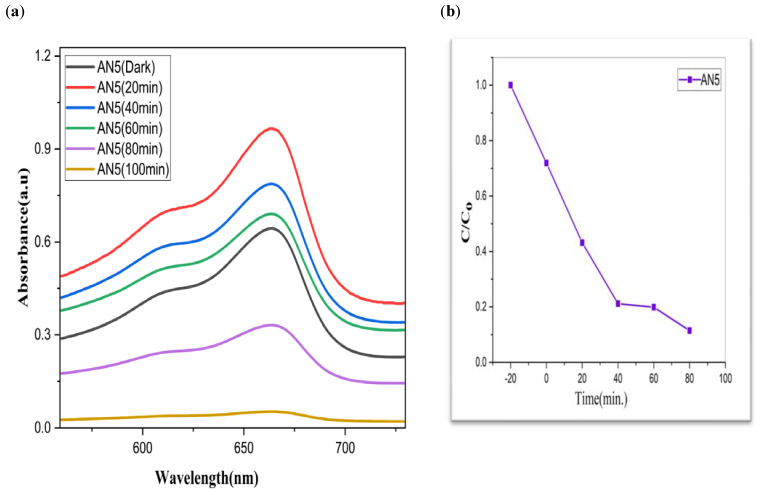

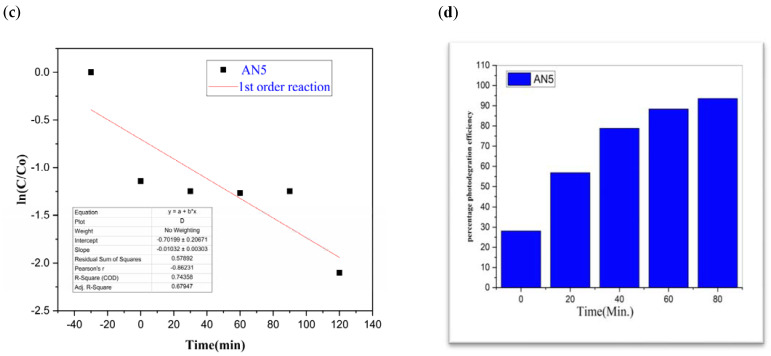
AN5 (**a**) graphical representation of degradation of methylene blue; (**b**) C/C_0_; (**c**) lnC/C_0_; (**d**) %age efficiency.

To investigate the photocatalytic degradation of organic dyes like methylene blue, nanoparticles of CdS, MoS_2_, and Bi_2_S_3_ were utilized. Figure 5 shows the typical degradation of methylene blue dye in the manifestation of CdS, MoS_2_, and Bi_2_S_3_, as well as the chemical and structural characteristics of the organic dyes used in this work [41]. Using lamps with wavelengths of 254 nm and 765 nm, ultraviolet light was used to expose the dyes. The Figure 7, Figure 8 and Figure 9 show a typical measurement of organic dye absorbance vs. reaction time after exposure to a UV-Vis lamp with a longer wavelength of 670 nm. In methylene blue, the hetero-poly aromatic bond between the S and N atoms accounts for the high absorbance at 664 nm [42,43].

Due to n to π* (the nitrogen atom has an unpaired electron pair) and to π* (a system of double bonds that are conjugated in aromatic rings), absorbance peaks at 664 and 615 nm indicated that an electronic transition was occurring in the MB solution [21,22].

The value of MB solution absorbance in the absence of UV light and the CdS/MoS_2_/Bi_2_S_3_ catalyst both dropped after 30 min, indicating that the CdS/MoS_2_/Bi_2_S_3_ catalyst has a strong degrading capability. When CdS/MoS_2_/Bi_2_S_3_ was utilized, the MB was completely damaged after 120 min of irradiation, but only 92% degraded after 100 min. Other researchers have reported comparable results using CdS/MoS_2_/Bi_2_S_3_ for photocatalytic MB breakdown (Figure 7, Figure 8 and Figure 9).

Although CdS/MoS_2_/Bi_2_S_3_ particles had higher photocatalytic activity, the composite has nearly two times more dye absorption under similar conditions, indicating that the CdS/MoS_2_/Bi_2_S_3_ phase had lower catalytic performance (Figure 7, Figure 8 and Figure 9).

Improved photocatalytic activity is anticipated from the anatase phase CdS/optimal MoS_2_’s size quantization effect, which is the consequence of a larger surface area and a larger band gap as a result of smaller particles. Though MB degradation effectiveness was discovered to be 90% in the contemporary analysis, which might be connected to the presence of more oxygen, as mentioned above, as well as smaller particle size obtained during CdS/MoS_2_/Bi_2_S_3_ production.

It has been advocated that the low electron hole pair recombination rate of semiconductor nanoparticles is the most important factor in their efficient photocatalytic activity. Due to electronic excitation inside “CdS/MoS_2_/Bi_2_S_3_”, the photocatalytic process is kicked off when nanocomposite “CdS/MoS_2_/Bi_2_S_3_” is exposed to UV light.

Direct activation of the nanoparticles is possible with energy superior to the bandgap of CdS, which is 3.2 eV (385 nm) [44,45]. According to numerous studies in the literature, the holes and electrons fashioned during photolysis can oxidize organic molecules, resulting in all of them being oxidized to ^•^OH radicals when they interact with water or hydroxides. Photogenerated electrons may also enter the dye or combine with absorbed O_2_ on nanoparticle planes to form the superoxide radical anion O_2_^•^ [46].

**Figure 10:**
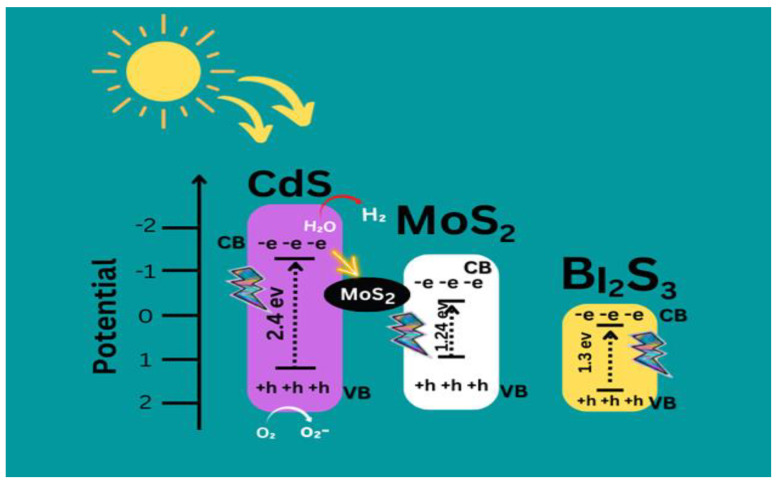
Proposed Mechanism of CdS/MoS_2_/Bi_2_S_3_.

The succeeding Equations (1)–(8) recapitulate the overall deterioration process as demonstrated graphically in Figure 10 [15].

So,

hv = photons,

h = holes and e = electrons

CdS/MoS_2_/Bi_2_S_3_ + hv (UV) → CdS/MoS_2_/Bi_2_S_3_ (eCB− + hVB+) (1)

CdS/MoS_2_/Bi_2_S_3_ (hVB+) + H_2_O → CdS/MoS_2_/Bi_2_S_3_ + H^+^ + ^•^OH (2)

CdS/MoS_2_/Bi_2_S_3_(hVB+) + OH^−^ →CdS/MoS_2_/Bi_2_S_3_ + ^•^OH (3)

CdS/MoS_2_/Bi_2_S_3_ (eCB−) + O_2_ → CdS/MoS_2_/Bi_2_S_3_ + O_2_^− •^ (4)

O^2− •^ + H ^•^ → HO^2•^ (5)

methylene blue dye + OH^•^ →degradation yields (6)

methylene blue dye + hVB+ → chemical by- yields of oxidation(7)

methylene blue dye + eCB^−^ → reducing substances (8)

examining methylene blue’s photocatalytic activity in its breakdown.

The photocatalytic activity of CdS/MoS_2_/Bi_2_S_3_ nanoparticles for dye degradation was tested utilizing extended UV irradiation. The photocatalytic activity of “CdS/MoS_2_/Bi_2_S_3_” against MB is shown in the figure. The activity was set up to be appropriate in both conditions, though the degradation efficiency was found to be lower when associated with short UV irradiation. Other dyes were also studied to investigate the ability of composites to degrade them when exposed to UV light over longer periods of time the results of which will be published in our future research.

The graphs demonstrate the degradation of MB dye in the presence of CdS/MoS_2_/Bi_2_S_3_. Photocatalytic experiments with various compositions of nanocomposites demonstrated that the highest photocatalytic efficacy of about 90% was found to be for MB dye. Especially the photocatalytic degradation efficiency of “CdS/MoS_2_/Bi_2_S_3_” was found to be maximum i.e., 91% at ration of 2%:5%:5% (Table 1).

While comparing, CdS has a 60% efficiency in the photodegradation of MB, whereas MoS_2_ only manages 19% (Table 1). But when we switched from Bi_2_S_3_ to CdS/MoS_2_, we saw a 90% boost in efficiency. The optimal outcome was obtained using a composition of CdS, MoS_2_, and Bi_2_S_3_. The “CdS/MoS_2_/Bi_2_S_3_” performed the best i.e., 91% (Table 1).

We looked at the photocatalytic deterioration of MB in the presence of visible light. Different molar ratios in “CdS/MoS_2_/Bi_2_S_3_” samples showed more significant photocatalytic activity when compared to dark blue “CdS/MoS_2_/Bi_2_S_3_”. The enhanced photocatalytic performance of “CdS/MoS2/Bi_2_S_3_” composite photocatalysts was also found. The “CdS/MoS_2_/Bi_2_S_3_” samples with microstructure also have a lot of potential applications in water treatment.

## 3. Materials and Methods

### 3.1. Synthesis of MoS_2_ Photocatalyst

**Figure 11:**
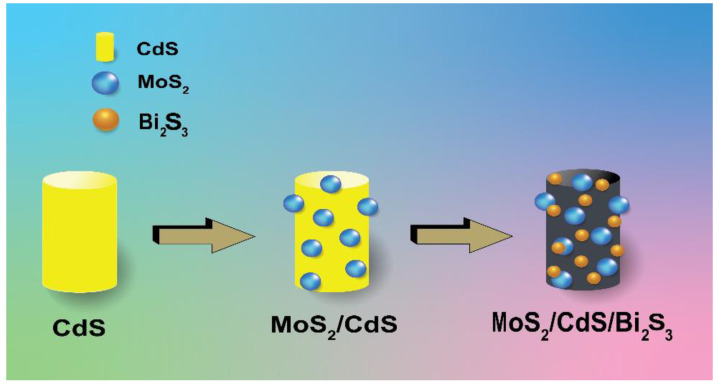
Product of CdS/MoS_2_/BiS_3_.

The graphical representation of the synthesis of photocatalyst composites has been shown in Figure 11. A 0.92 g sample of ammonium heptamolybdate tetrahydrate was diluted in 50 mL of ethylene glycol. Then, it was continuously mixed with 5 mL of hydrazine for 30 min. The mixture was then heated to 200 °C for 24 h while enclosed in a hydrothermal reactor coated with Teflon. After the reaction was complete, the container was allowed to cool at room temperature, and distilled water was used to wash the product. After multiple washes in ethanol, the resulting material was placed into a heated oven at 60 °C for 24 h in order to dehydrate. The product was allowed to cool at room temperature until further characterization and photocatalytic investigation.

### 3.2. Synthesis of CdS Photocatalyst

In the first step, 3.085 g of “Cd(NO_3_)_2_•4H_2_O” cadmium nitrate tetrahydrate was dissolved in 25 mL of distilled water. After that, 1.565 g of “Na_2_S” and 0.316 g of “Na_2_SO_3”_ were added to the aqueous solution of cadmium nitrate tetrahydrate. A 5 mL solution of acetic acid, 20 mL of ethylene glycol, and 1 g of “Na_2_EDTA” were then added with constant stirring for 30 min. Furthermore, the solutions were mixed well in a stainless-steel autoclave lined with Teflon, which was then sonicated for an hour. After that, the autoclave was cooked in an air-blowing thermostatic oven at 180 °C for six hours. The residue was dried in a vacuum chamber at room temperature overnight, followed by washing with ethanol and water.

### 3.3. Synthesis of CdS/MoS_2_/Bi_2_S_3_ Composites

**Figure 12:**
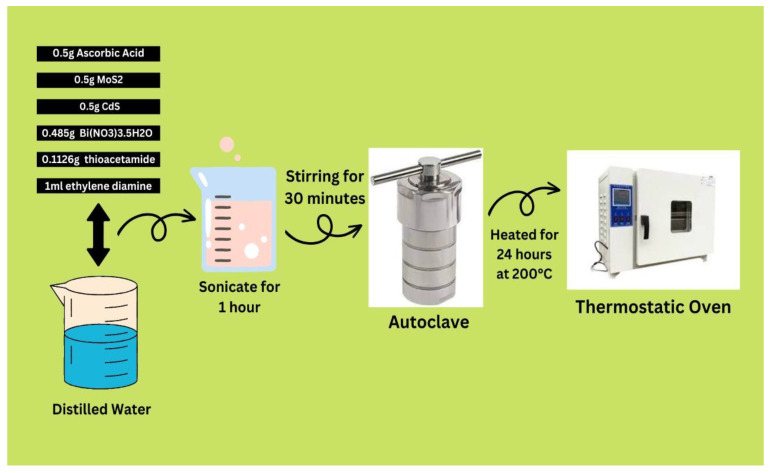
Synthesis of CdS/MoS_2_/Bi_2_S_3_ composites.

To obtain the wanted solution, we took 0.5 g of ascorbic acid dissolved in distillation water, added 0.5 g of MoS_2_, 0.5 g of CdS, 0.485 g of Bi(NO_3_)_3_.5H_2_O as a source of Bi, and 0.1126 g of thioacetamide as a source of sulfur, followed by 1 mL of ethylene diamine and 30 min of stirring (Figure 12). This solution was sonicated for 1 h after being well mixed in a Teflon-lined stainless steel autoclave. The autoclave, formed of stainless steel and layered with Teflon material, was placed in a thermostatic oven that heated the air to 200 °C for 24 h. After being washed in ethanol and water, the residue was dried in a vacuum compartment at room temperature for 12 h. We synthesized three different composites by varying the ratios of CdS, MoS_2_, and Bi_2_S_3_. The synthesized composites with specific ratios were as follows: AN3 with a ratio of 1:1:1; AN4 with a ratio of 1%/5%/5%; and AN5 with a ratio of 2%/5%/5% of CdS, MoS_2_, and Bi_2_S_3_, respectively.

## 4. Conclusions

We have successfully made “CdS/MoS_2_/Bi_2_S_3_” nanohybrids with different structures and looked at their photodegradation processes. The 3D designs of “CdS/MoS_2_/Bi_2_S_3_” nanohybrids displayed unusual sunlight-induced photocatalytic activities for decomposing three organic compounds. In sunlight irradiation, the “CdS/MoS_2_/Bi_2_S_3_” nano heterojunctions found extraordinarily high photodegradation efficiencies, resulting in the breakdown of 90.3% of the MB dye within 120 min. Superior photodegradation activity has been attained because of the synergistic effect’s high density between “CdS/MoS_2_” and Bi_2_S_3_. This effectively reduces the recombination rate and increases the charge separation process. Significant data demonstrate how varied “CdS/MoS2/Bi_2_S_3_” nanohybrid morphologies impact the heterojunction density. This work emphasizes the straightforward method for creating “CdS/MoS_2_/Bi_2_S_3_” heterojunctions for the outstanding photodegradation of organic azo dyes and pharmaceutical waste driven by sunlight. It can be shown that the cost-effective “CdS/MoS_2_/Bi_2_S_3_” nanohybrids are particularly useful for several industrial applications, including H_2_ production, solar cells, and water splitting.

## Figures and Tables

**Table 1:** Degradation %age efficiency of synthesized materials.

Sr. No.	Composite	Percentage	Photodegradation Efficiency
1	MoS_2_	-	19%
2	CdS	-	65%
3	CdS/MoS_2_/Bi_2_S_3_	1:1:1	90%
4	CdS/MoS_2_/Bi_2_S_3_	1:5:5	88%
5	CdS/MoS_2_/Bi_2_S_3_	2:5:5	91%

## Data Availability

The data will be available on request.

## Sample Availability

Samples of the compounds are available from the authors.
